# Safety, tolerability, pharmacokinetics, and pharmacodynamics of low dose lysergic acid diethylamide (LSD) in healthy older volunteers

**DOI:** 10.1007/s00213-019-05417-7

**Published:** 2019-12-18

**Authors:** Neiloufar Family, Emeline L. Maillet, Luke T. J. Williams, Erwin Krediet, Robin L. Carhart-Harris, Tim M. Williams, Charles D. Nichols, Daniel J. Goble, Shlomi Raz

**Affiliations:** 1 Eleusis Benefit Corporation, New York, NY USA; 2 Imperial College London, London, UK; 3 Bristol University, Bristol, UK; 4 Department of Pharmacology and Experimental Therapeutics, LSU Health Sciences Center, New Orleans, LA USA; 5 San Diego State University, San Diego, CA USA

**Keywords:** Inflammation, Serotonin, CNS, Neurodegenerative disease, Psychedelics, Clinical trial, Alzheimer’s, Immune system, 5-HT_2A_

## Abstract

**Abstract:**

Research has shown that psychedelics, such as lysergic acid diethylamide (LSD), have profound anti-inflammatory properties mediated by 5-HT_2A_ receptor signaling, supporting their evaluation as a therapeutic for neuroinflammation associated with neurodegenerative disease.

**Objective:**

This study evaluated the safety, tolerability, pharmacokinetics, and pharmacodynamics of orally repeated administration of 5 μg, 10 μg, and 20 μg LSD in older healthy individuals. In the current paper, we present safety, tolerability, pharmacokinetics, and pharmacodynamic measures that relate to safety, tolerability, and dose response.

**Methods:**

This was a phase 1 double-blind, placebo-controlled, randomized study. Volunteers were randomly assigned to 1 of 4 dose groups (5 μg, 10 μg, 20 μg LSD, and placebo), and received their assigned dose on six occasions (i.e., every 4 days).

**Results:**

Forty-eight older healthy volunteers (mean age = 62.9 years) received placebo (*n* = 12), 5 μg (*n* = 12), 10 μg (*n* = 12), or 20 μg (*n* = 12) LSD. LSD plasma levels were undetectable for the 5 μg group and peak blood plasma levels for the 10 μg and 20 μg groups occurred at 30 min. LSD was well tolerated, and the frequency of adverse events was no higher than for placebo. Assessments of cognition, balance, and proprioception revealed no impairment.

**Conclusions:**

Our results suggest safety and tolerability of orally administered 5 μg, 10 μg, and 20 μg LSD every fourth day over a 21-day period and support further clinical development of LSD for the treatment and prevention of Alzheimer’s disease (AD).

**Electronic supplementary material:**

The online version of this article (10.1007/s00213-019-05417-7) contains supplementary material, which is available to authorized users.

## Introduction

Lysergic acid diethylamide (LSD) is a classic serotonergic hallucinogenic (psychedelic) drug from the ergoline family. LSD has a complex polypharmacology, interacting with essentially all aminergic G protein–coupled receptors (GPCRs) (Wacker et al. 2017). However, its defining psychoactive properties are mediated primarily through the serotonin 2A (5-HT_2A_) receptor, and are specifically blocked by 5-HT_2A_ receptor antagonists like ketanserin (Preller et al. 2017, 2019).

Alzheimer’s disease (AD) is a devastating neurodegenerative condition without effective disease-modifying treatments. Symptomatic hallmarks of AD include memory loss, cognitive impairment, and neuropsychiatric disorders such as apathy, anxiety, and depression. Memory loss and cognitive impairment correlate with synaptic dysfunction in AD (Coleman et al. 2004), which has been hypothesized to be the result of the toxic accumulation of soluble oligomers of the amyloid beta peptide (AβOs) (Ferreira and Klein 2011).

Converging lines of evidence suggest that the 5-HT_2A_ receptor is a promising therapeutic target in AD. 5-HT_2A_ receptors are significantly involved in mediating memory and cognition (Zhang and Stackman 2015; Harvey 2003; Romano et al. 2010), and impairment of these functions in AD is accompanied by serotonergic denervation in associated brain regions (Geldenhuys and Van der Schyf 2011; Ramirez et al. 2014). Specifically, pathological changes in the highly serotonergic dorsal raphe nucleus, observed in the earliest stages of AD, appear to significantly influence the symptoms and pathogenesis of AD (Šimić et al. 2016; Grinberg et al. 2009). While 5-HT_2A_ receptor density generally decreases with age, significant declines in 5-HT_2A_ receptor binding are associated with depression in older patients and rapid loss of 5-HT_2A_ receptors in the cortex precedes diagnosis of mild cognitive impairment. Furthermore, the rate of 5-HT_2A_ receptor loss is correlated to the rate of cognitive decline in AD patients (Hasselbalch et al. 2008; Marner et al. 2012; Sheline et al. 2004).

Serotonergic system dysfunction is also implicated in the neuropsychiatric symptoms of AD, including apathy, anxiety, and depression; depressive symptoms in cognitively normal older adults are associated with AD-like neurodegeneration of serotonergic regions of the brain independent of amyloid burden (Geerlings et al. 2008; Lyketsos and Olin 2002; Donovan et al. 2015). A polymorphism of the gene encoding the 5-HT_2A_ receptor has been correlated with susceptibility to depressive symptoms in AD (Holmes et al. 2007). Recent clinical trials have demonstrated potent and enduring anti-depressive and anxiolytic effects of classical psychedelic drugs psilocybin and LSD (Gasser et al. 2014; Carhart-Harris et al. 2016; Horstmann et al. 2010; Ross et al. 2016; Griffiths et al. 2016; Grob et al. 2011). Several studies have highlighted the mystical nature of the subjective experience resulting from administration of high dose psilocybin or LSD as a mediator for the psychiatric therapeutic benefits (Russ et al. 2019; Garcia-Romeu et al. 2014; Roseman et al. 2018; Haijen et al. 2018). However, recent evidence of sustained antidepressant-like effects of psilocybin in an animal rodent model for the study of depression after only a single dose suggests that solely biological mechanisms dependent on 5-HT_2A_ receptor activation may effectively underlie key CNS effects observed for this class of drug (Hibicke et al. 2019).

The 5-HT_2A_ receptor’s involvement in immunomodulation is also relevant to its potential as a therapeutic target in AD. Neuroinflammation is a key pathological hallmark of AD, accelerating the pathogenesis of AD through feedback loops between inflammation and amyloid precursor protein (APP) processing, leading to elevated and continuous AßO aggregation and disturbance of microglial and immune function (Ledo et al. 2016; Heneka et al. 2015). 5-HT_2A_ receptor agonists modulate microglial function (Glebov et al. 2015; Krabbe et al. 2012), and higher 5-HT_2A_ receptor expression is found in reactive astrocytes of AD patient brain tissue (Wu et al. 1999). Serotonin (5-HT) itself blocks AßO-induced microglial activation in vitro (Ledo et al. 2016), suggesting that direct 5-HT_2A_ receptor agonism may have the capacity to normalize chronic pathogenic microglial activation. Furthermore, the neuroprotective and neurotrophic effects of a number of 5-HT_2A_ receptor agonists have been observed in a human neuronal cell line (SK-N-SH) model as well (Marinova et al. 2013, 2017).

LSD and other psychedelic agonists of the 5-HT_2A_ receptor are proposed as modulators of pathogenic inflammation and APP processing loops. Historically, serotonin-mediated 5-HT_2A/C_ receptor agonism was shown to promote non-amyloidogenic processing of APP in 3T3 cells stably overexpressing these receptors (Nitsch et al. 1996). More recently, studies in cell culture models (Yu et al. 2008) and whole animals (Nau et al. 2013, 2015) have demonstrated that activation of 5-HT_2A_ receptor by psychedelics, such as LSD, produces potent anti-inflammatory effects at non-behaviorally active doses (see also Nichols 2016). Of note, chronic administration of LSD was observed to reduce cortical soluble Aß40 and Aß42 protein expression in a McGill-R-Thy1-APP AD rat model (Shlomi Raz, personal communication).

These findings support further evaluation of psychedelics in the treatment and possible prevention of AD. The ability of LSD to easily cross the blood–brain barrier (BBB) and potentially suppress chronic neuroinflammation makes it a particularly suited centrally acting drug candidate, given the clinical failure of non-BBB penetrant conventional non-steroidal anti-inflammatory drugs (NSAIDs) (ADAPT Group 2013) and TNF-α inhibiting monoclonal antibody therapies (Butchart et al. 2015).

The chief therapeutic limitation of LSD and other psychedelics is their behavioral liability and psychoactivity. This liability is dose-dependent, however, and anti-inflammatory effects associated with psychedelics may be therapeutically applicable at sub-perceptual doses in humans as they are in animal models (Yu et al. 2008; Nau et al. 2013). Furthermore, a number of anecdotal uncontrolled reports associate the ingestion of sub-perceptual doses, or “microdoses” as called by some, of LSD, with enhanced mood and cognition (Fadiman 2011; Kuypers et al. 2019). Although these reports must be viewed with caution as they are uncontrolled (Friedman 2017), they suggest possible therapeutic effect in cognitive decline associated with AD at sub-perceptual dose levels of LSD.

In a recent study, healthy young adults were administered placebo, 6.5 μg, 13 μg, and 26 μg LSD in randomized order at 1-week intervals (Bershad et al. 2019). In this within-subject design, LSD produced dose-dependent subjective effects. After the 26 μg, participants reported increased “vigor” and slightly decreased positivity ratings of positive emotional images. All other measures of mood, cognition, and physiology were unaffected. Furthermore, the researchers found that 13 μg is a threshold dose.

The primary objectives of our study were to determine if periodic administration of 5 μg, 10 μg, or 20 μg of LSD can be tolerated without impairment to normal cognition and disruption of typical daily activity in a population of older healthy volunteers aged between 55 and 75 years, and to evaluate pharmacokinetics (PK). In addition, the study sought to evaluate pharmacodynamics and dose-response relationships. Dose selection was guided by research indicating that the lowest perceptible dose of LSD is between 10 and 20 μg, with doses at or below these levels associated with minimal changes in perception, cognition, and affect (Greiner et al. 1958). Periodic dosing over 3 weeks (6 doses, administered every 4th day) was selected based on a regimen proposed based on anecdotal reports (Fadiman 2011).

A subset of results from this study was published separately, from a task exploring the effects of 5 μg, 10 μg, and 20 μg of LSD on time perception (Yanakieva et al. 2018). Ratings on a regularly completed subjective drug effects questionnaire were used to investigate any impact of peak times of self-perceived drug effects on interval timing. They reported descriptive data for five subjective effect questions, including “do you feel high,” “do you feel a drug effect,” and “do you feel your ability to concentrate is the same, better, or worse than normal.” This analysis revealed that LSD conditions were not associated with any robust changes in self-report indices of perception, mentation, or concentration. However, despite the lack of perceptual effects, LSD reliably produced over-reproduction of temporal intervals of 2000 ms and longer with these effects most pronounced in the 10 μg dose condition. This offers the interpretation that sub-perceptual doses of LSD may cause changes in fundamental cognitive systems, such as attention and working memory.

Here, we report safety measures and pharmacokinetics. While detailed results on exploratory pharmacodynamic assessments are beyond the scope of this paper, we report selected validated cognitive, sensory, and motor measures that may reveal differences in dose groups.

## Methods

This was a phase 1, double-blind, placebo-controlled, randomized study of repeat dosing of 5 μg, 10 μg, and 20 μg of LSD. The primary objectives were to evaluate the safety, tolerability, and pharmacokinetics of low dose LSD. Given the stage of development, this investigation aimed to explore a range of secondary outcomes related to cognition, sensory, and motor effects; a number of which are reported here.

The study was conducted in accordance with Good Clinical Practice, as required by the United Kingdom Statutory Instrument 2004 No.1031, The Medicines for Human Use (Clinical Trials) Regulations, and subsequent amendments. It was also performed in accordance with the ethical principles that have their origin in the Declaration of Helsinki. The study protocol and informed consent form were reviewed and approved by the independent ethics committee for the investigational site in accordance with International Conference on Harmonisation harmonized tripartite guideline on Good Clinical Practice and UK law. Each volunteer provided written informed consent after adequate explanation of the aims, methods, anticipated benefits, and potential hazards of the study.

### Study volunteers

Healthy volunteers aged 55 to 75 years were screened within 28 days before randomization. Volunteers were assessed for eligibility based on medical history, physical examination, and inclusion and exclusion criteria. The key inclusion criterion was no LSD use in the past 5 years. Based on the modified SCID-CT, a subject with the lifetime presence of any of the following was excluded: psychotic symptoms that are not substance-induced or due to a medical condition or has a first- or second-degree relative with these disorders; any manic or hypomanic episode; lifetime presence of any major depressive episode; lifetime presence of substance abuse, or dependence on any substance in the past 5 years; current diagnosis of obsessive-compulsive disorder (OCD), dysthymic disorder, panic disorder, anorexia, and bulimia. Also excluded were volunteers who were current smokers, had blood pressure exceeding 160 mmHg (systolic) and 90 mmHg (diastolic), or were receiving concurrent medication or herbals intended for (or off label) psychiatric disorders or conditions, such as those mentioned above and non-exhaustively. In practice, none of the participants received concomitant CNS (central nervous system) medications during the trial period, except for one subject who received zopiclone for occasional insomnia in the placebo group. Volunteers were administered the Columbia-Suicide Severity Rating Scale (C-SSRS) and the SCID-CT (Clinical Trials Version; First et al. 2007) as a screening tool, modified based on guidelines described by Johnson et al. (2008) regarding human hallucinogen research.

### Study design

A total of 48 subjects were included in the study. For logistical purposes, the investigation was conducted in 4 separate cohorts of 12 subjects each. Volunteers were assigned to 1 of 4 cohorts then randomly assigned to 1 of 4 dose groups: 5 μg, 10 μg, or 20 μg LSD of the active moiety or placebo in a 1:1:1:1 ratio within each cohort. Volunteers and study personnel were blinded to dose received but were aware of the dose range under investigation.

Volunteers received their assigned study dose in an in-patient setting on 6 separate occasions over 21 days with a 96-h interval. Volunteers were administered the same dose on each occasion, and each dosing day was spaced by 3 non-dosing days (i.e., doses were administered on days 1, 5, 9, 13, 17, and 21). All volunteers were kept at the clinical site for approximately 8–12 h post-dosing for testing, blood sampling, and safety monitoring. A total of 32 volunteers (i.e., 8 from each drug group) provided data for the evaluation of the pharmacokinetics (PK) on dose 1 and dose 6. A follow-up visit was conducted approximately 4 weeks after the last dose.

d-Lysergic acid diethylamide (d-LSD, HPLC purity > 99%, Onyx Scientific Limited, UK) was dissolved in ethanol at 25 mg/mL and prepared as a solution 50 μg or 2 μg d-LSD/mL in distilled water and completed to a final volume of 10 mL distilled water for oral administration. Placebo was distilled water only and was presumably indistinguishable from the LSD solution, especially given the very low dose.

### Safety and tolerability assessments

Safety and tolerability evaluations consisted of adverse event (AE) monitoring, administration of the C-SSRS, blood pressure, and pulse rate at every visit from screening through to follow-up. Clinical laboratory evaluations included hematology, blood chemistry, and urinalysis at screening, baseline, doses 3 and 6, and follow-up. Electrocardiogram (ECG) parameters and physical examinations were assessed at screening and follow-up.

Treatment Emergent Adverse Events (TEAEs) that occurred in more than 2 volunteers are reported, and the incidence of AEs reported in more than 10% of volunteers was analyzed using a logistic regression model.

### Pharmacodynamic assessments

Pharmacodynamic assessments reported here focus on validated cognitive tests, measurements of balance and proprioception, and subjective drug effect questionnaires. Other exploratory pharmacodynamic endpoints might be presented in future communications. No formal statistical assessment of sample size was conducted, but the number of volunteers was considered sufficient for this stage of development.

### Cognition assessments

The Cambridge Neuropsychological Test Automated Battery (CANTAB) (Cambridge Cognition 2016) was performed at screening, baseline, and doses 3 and 6, between 2 and 3 h after dose administration and at the follow-up visit. Screening results were not included in analyses, as this time point was used only for familiarization of the volunteers to the battery. CANTAB tests were administered via iPad and included the following tasks:

Reaction time (RTI) measures reaction times to a visual target that appears on the screen.

Paired associates learning (PAL) measures visual memory and learning. Six boxes are presented on the screen. The volunteer must remember the pattern under each card and respond when prompted at the end of the trial.

Rapid visual information processing (RVP) measures visual attention. The volunteer is presented with single digits appearing sequentially in the center of the screen. The task is to look for a particular sequence of 3 numbers presented on the top right corner of the screen. When the sequence appears, the volunteer must tap a button.

Spatial working memory (SWM) measures spatial working memory. The volunteer is presented with boxes on the screen and is asked to find tokens that are hidden behind the boxes. The tokens can be revealed by touching the boxes and if a token is found in a box, that box will not be used again to hide other tokens in the same trial.

The CANTAB assessments resulted in 56 endpoints for analysis. Assessment scores were used to yield descriptive statistics for the 5 μg, 10 μg, 20 μg, and placebo groups at baseline, doses 3 and 6, and follow-up. Comparisons between treatment groups were performed using a one-way ANOVA with Bonferroni post hoc tests for each endpoint across time points similar to the CANTAB analysis method described in (Kuzmickienė & Kaubrys, 2015).

### Balance and proprioception

Balance was assessed at screening, approximately 4 h post-dosing at every dose, and at follow-up. BTrackS™ is a balance measurement system, which computes postural sway based on a volunteer’s center of pressure (Goble et al. 2017; O’Connor et al. 2017).

Proprioception, the ability to sense stimuli arising within the body regarding position, motion, and equilibrium, was assessed at baseline and follow-up; and 2–3 h post-dosing at two of the following four doses: 2, 3, 4, and 5. The proprioception protocol is summarized in Goble (2010).

Balance and proprioception measures were analyzed using a repeated measures mixed-model (RMMM) where assessments were performed over time. The dependent variable in the model was the change from baseline value. The independent variables included dose (5 μg, 10 μg, 20 μg, or placebo) as a fixed factor, volunteers as a random term, and time (i.e., baseline, doses 1–6, follow-up) as a repeated term. The baseline value was used as a covariate term in the model to control for variable baseline performance.

### Drug effect assessments

Subjective drug effect questions can be used to assess perceptual alterations and tolerability of a drug. A 22-question assessment was administered during dose days at multiple time points via visual analog scale (VAS). Time points were 15 min pre-dose and then at 0.5, 1, 1.5, 2, 2.5, 3, 4, 5, 6, 7, and 8 h post-dosing. The VAS consisted of 22 questions drawn from different sources that include drug abuse liability measures: *Drug Effects Questionnaire* (Shram et al. 2011); *Addiction Research Centre Inventory* (Haertzen et al. 1963); *Subjective Effects of Substances with Abuse Potential* (Farré et al. 2007); and subjective scales previously used to investigate LSD (Schmid et al. 2014). Volunteers were presented with one VAS question at a time, with a slider allowing continuously varying response from 0 to 100%.

The Five Dimensional Altered States of Consciousness (5D-ASC) questionnaire is a self-report questionnaire used to measure retrospectively subjective experiences of altered states of consciousness. The 94 items were presented on a VAS scale (Dittrich 1998) and completed at approximately 7 h post-dose at every dose. The original five dimensions on which this scale measures alterations in consciousness include the following: oceanic boundlessness, dread of ego dissolution, visionary restructuralization, vigilance reduction, and auditory alterations. Changes were also assessed on the following eleven new subscales (Studerus et al. 2010): experience of unity, spiritual experience, blissful state, insightfulness, disembodiment, impaired control and cognition, anxiety, complex imagery, elementary imagery, audio-visual synesthesia, and changed meaning of percepts.

Both assessments were completed on Psytools (Delosis, London) via laptop computer.

For the VAS of subjective effects, maximum value (*E*_max_) was used to assess differences across drug groups. *E*_max_ represents the peak effects measured in the day (i.e., a single time point). The dose-response relationship for subjective effects (22 variables) and 5D-ASC (16 variables) was explored using polynomial regression models with linear, quadratic, and cubic terms. For the VAS, the *E*_max_ values were averaged across dose days for each question, and for the 5D-ASC, the average value was over six doses for each of the variables.

### Pharmacokinetics assessments

#### Blood sampling

Blood samples for plasma analysis of LSD were collected within 15 min prior to dosing and at 30 min and 1, 2, 4, 8, and 12 h post-dosing. Post-dose samples were taken ± 10% of scheduled time or ± 4-h window around the scheduled time, whichever was narrower. Immediately after blood collection, two lithium heparin tubes (1 × 4 mL and 1 × 6 mL, BD Diagnostics, Vacutainer®) were kept on wet ice until centrifuged. Within 30 min of blood collection, samples were separated by centrifugation for 10 min (1000–1200 g) at approximately 4 °C. The resultant plasma was withdrawn in approximately equal volumes of 1.5 mL into 2 appropriately labeled polypropylene tubes for PK assay and stored (within 1 h of the sampling time) at − 80 °C or lower until shipment.

#### Drug levels determination

Drug level determination in human plasma samples was performed at Analytical Services International Ltd. (UK).

For quantification, the LC-MS/MS system used consisted of an Applied Biosystems API4000 mass spectrometer coupled to a Agilent 1100 Series Micropump HPLC system. The HPLC system was equipped with an Agilent 1100 Series autosampler.

LSD and the internal standard (IS) LSD-D3 were separated on a 150 × 2.1 mm Alltima™ C18 5 μm stainless steel column (Grace). Mobile phase A consisted of methanol and mobile phase B was 10% methanol:formic acid solution, with a flow rate of 1.0 mL/min. The detector was an Applied Biosystems API4000 Turbo Ion Spray using Positive Ion Mode. The preparation of the calibration curve and calculation of plasma concentrations used least squares linear regression analysis with weighting factor l/× 2 obtained by the internal standard method using peak area. Computer software, Analyst 1.3.2, was used for chromatogram data analysis and quantitative calculation.

Stock solutions of LSD and IS were prepared by dissolving the accurately weighed drug in 0.1% formic acid water at concentrations of 10 μg/mL. Working solutions of LSD were freshly prepared by serially diluting the stock, to a calibration range of 200 pg/mL to 10 ng/mL, in blank control serum. A total of 100 μL plasma calibrator/control/samples was added to corresponding 5-mL polypropylene tubes. A total of 100 μL internal standard solution, 500 μL sodium hydrogen carbonate solution (saturated), and 2 mL of MTBE was added to all tubes. All tubes were mixed for a minimum of 15 min and centrifuged for a minimum of 2 min at 3500 rpm. The supernatant was transferred to clean 5-mL polypropylene tubes and evaporated to dryness in a SpeedVac. The extracts were reconstituted with 250 μL of 10% methanol/formic acid solution and allowed to stand for a minimum of 5 min before being briefly vortex-mixed. The extracts were transferred to polypropylene autosampler tubes for analysis by LC MS/MS.

#### Pharmacokinetic analysis

The PK parameters were derived, using non-compartmental methods, from plasma drug concentrations over time profiles for each individual volunteer. Maximum measured concentration *C*_max_ and peak time *T*_max_ were obtained directly from concentrations over the 12-h time profiles, as well as *T*_first_ and *T*_last_ (i.e., first and last quantifiable drug levels during the observation period). The lower limit of quantification (LLOQ) of LSD was 200 pg/mL. *λ*_*z*_ (i.e., terminal elimination rate constant) was calculated by linear regression of the terminal linear portion of the loge concentration vs. time curve with a 1/Y^2^ weighting method, and *t*_1/2_ (i.e., elimination half-life) was derived as ln(2)/*λ*_*z*_. The AUC_0-t_ was calculated using trapezoidal summation of the plasma measurable concentrations over the observation period. AUC_0-inf_ was extrapolated from AUC_0-last_ and the terminal elimination rate constant *λ*_*z*_ as: AUC_0-inf_ = AUC_0-t_ + *C*_last_/*λ*_*z*_(m), where *C*_last_ was the last measurable concentration and *λ*_*z*_(m) was the grand mean of all calculated *λ*_*z*_ (here, 0.085). Recorded drug levels below the level of quantification (LLOQ), which included all values under 200 pg/mL, were treated as missing and excluded except for those recorded pre-dosing (time 0 h). For the purpose of calculating AUC_0-t_ when two consecutive plasma concentrations below LLOQ were encountered after *T*_max_, all subsequent values were excluded from the analysis.

Individual plasma concentrations of LSD parent drug were summarized by dose group (LSD 5 μg, 10 μg, and 20 μg and placebo), following dose 1 and dose 6 using descriptive statistics of the arithmetic mean, standard deviation, coefficient of variation (CV%), geometric mean, median, minimum and maximum, and number of observations. With 8 volunteers per treatment dose group, the form of the relationship between the administered dose and the extent of absorption (AUCs, *C*_max_) was also investigated following both dose 1 and dose 6.

## Results

### Volunteer demographics

Of 96 screened volunteers, 48 were determined eligible. In total, 27 male and 21 female volunteers with an average age of 62.9 years and body mass index (BMI) of 27.3 enrolled in the study. Age, gender, and BMI were balanced across dose groups. The demographic and baseline characteristics for each dose group are summarized in Table 1.

**Table 1:** Demographic and baseline characteristics of volunteers

Observation		5 μg	10 μg	20 μg	Placebo
		*N* = 12	*N* = 12	*N* = 12	*N* = 12
Age (years)		63.5 (SD = 6.29)	63.17 (SD = 4.8)	61.58 (SD = 6.64)	63.42 (SD = 5.2)
BMI (kg/m^2^)		26.7 (SD = 4.61)	28.79 (SD = 3.32)	26.63 (SD = 4.76)	27.09 (SD = 3.74)
Gender	Female (*n*)	6	6	3	6
	Male (*n*)	6	6	9	6
Race	Asian (*n* (%))	1 (8.3%)	0	1 (8.3%)	0
	Black (*n* (%))	1 (8.3%)	1 (8.3%)	0	2 (16.6%)
	White (*n* (%))	10 (83.3%)	11 (91.7%)	11 (91.7%)	10 (83.3%)

### Safety and tolerability

Clinical review revealed no difference between active and placebo groups and among the active dose groups in the proportion of AEs, and none was severe in intensity. LSD was well tolerated, and the frequency and intensity of adverse events were similar to placebo. No volunteer discontinued due to an AE and no unexpected AEs occurred.

Similarly, no clinically significant abnormalities based on vital signs, physical examinations, ECG measurements, and laboratory results were found in clinical review. The C-SSRS and psychiatrist interview, administered daily, revealed that no volunteer developed suicidal ideation and that all volunteers were recommended for release.

The overall rate of treatment–emergent AEs (TEAEs) was high, ranging from 66.7 to 83.3% of the participants in each group. All TEAEs that occurred are presented in Table 2.

**Table 2:** Treatment Emergent Adverse Events, listed by system organ class and preferred term

System organ class and preferred term	Placebo, *n* (freq)	5 μg, *n* (freq)	10 μg, *n* (freq)	20 μg, *n* (freq)
Ear and labyrinth disorders			1(1)	1(1)
Hyperacusis			1(1)	1(1)
Gastrointestinal disorders	2(6)	3(8)	2(2)	1(2)
Diarrhea	2(2)	1(1)	1(1)	
Nausea	1(2)	1(6)		1(1)
General disorders and administration site conditions	6(13)	4(11)	4(12)	7(10)
Asthenia	1(2)	2(3)		1(1)
Energy increased	3(4)	1(3)		1(1)
Fatigue	3(3)	2(3)	3(4)	2(2)
Feeling abnormal		2(2)		1(1)
Feeling hot	1(2)		1(5)	1(1)
Feeling of relaxation	2(2)			1(1)
Hunger			2(3)	1(1)
Metabolism and nutrition disorders	2(1)	2(4)		1(1)
Decreased appetite	1(1)	2(3)		
Increased appetite		1(1)		1(1)
Musculoskeletal and connective tissue disorders	1(1)	1(1)	1(1)	1(1)
Limb discomfort	1(1)		1(1)	
Nervous system disorders	8(18)	7(31)	8(21)	5(19)
Disturbance in attention	1(1)	1(1)	1(1)	
Dizziness	2(2)	5(7)	2(2)	2(3)
Headache	1(1)	2(9)	6(10)	3(11)
Hypersomnia	2(2)	1(1)	1(1)	
Lethargy		2(2)		1(1)
Somnolence	3(8)	5(8)	3(6)	2(4)
Psychiatric disorders	4(13)	1(8)	5(13)	4(6)
Abnormal dreams		1(1)	1(1)	
Elevated mood	2(4)	1(4)	1(1)	1(1)
Illusion	1(2)		1(2)	1(2)
Insomnia	2(2)	1(1)	2(4)	
Mood altered	1(1)		1(1)	
Sleep disorder	3(3)		1(1)	

The only difference noted in clinical review between placebo and the active treatment groups was frequency of headaches in the active treatment groups (16.7%, 50.0%, and 25.0% of volunteers at LSD doses 5 μg, 10 μg, and 20 μg, respectively) than in the placebo group (8.3%). All headaches were either mild or moderate in intensity.

### Pharmacokinetics

**Fig. 1:**
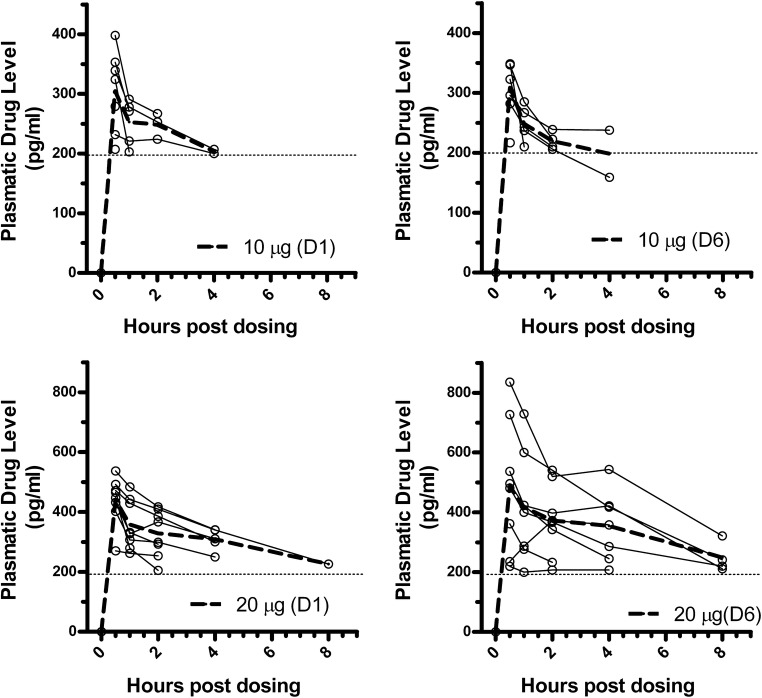
Dose 1 (left) and dose 6 (right) plasma concentrations of LSD for each volunteer after 10 μg LSD (*N* = 7) and 20 μg LSD (*N* = 8). Trace of the mean per dosing group from baseline to 8 h post-dose is represented by the dotted line

Drug levels were quantifiable for the 10 μg and 20 μg LSD conditions in average until the 4-h sampling time points after dose 1 (single dose) and dose 6 (after repeated doses). Drug levels were below LLOQ (200 pg/mL) at all sampled time points for either dose 1 or dose 6 after drug administration of 5 μg LSD, and for 1 of the 8 volunteers in the 10 μg LSD group. Plots of concentration in pg/mL by participant are presented in Fig. 1 for both doses.

Drug levels returned to below LLOQ prior to the last sampling time for each volunteer and each visit. Also, prior to administration of dose 6, drug levels were below LLOQ in all dose groups pre-dose.

The PK parameters AUC_0–4_, AUC_0-inf_, *C*_max_, and *T*_max_ are summarized for LSD 10 μg and 20 μg in Table 3 following doses 1 and 6. The *C*_max_ for the 10 μg LSD group was 305 ± 68 pg/mL for dose 1 and 309 ± 48 pg/mL for dose 6 with a peak time at 0.5 h. The *C*_max_ for the 20 μg LSD group was 440 ± 79 pg/mL for dose 1 and 504 ± 200 pg/mL for dose 6 with a peak time at 0.5 and 0.7 h, respectively. Mean *C*_max_, AUC_0–4_, and AUC_0-inf_ were well reproduced between doses 1 and 6, indicating these parameters were unaffected by repetitive dosing at 10 μg and 20 μg of LSD. The ratio between 10 μg and 20 μg dosing groups for *C*_max_ and AUC_0–4_ averaged at 1.5 to 1.7 for each dosing event, indicating a positive drug dose to plasma level relationship but deviating from linearity at these low doses. The estimation and accuracy of calculated *t*_1/2_ was limited by low plasmatic drug levels and incomplete data sets at time points after 4 h for most volunteers. The average terminal half-life across all data set was 8.25 ± 7.5 h and similar to the reported terminal half-life value of 8.9 ± 5.9 h of an oral dose of 200 μg of LSD (Dolder et al. 2015).

**Table 3:** Pharmacokinetic parameters summary for 10 μg and 20 μg following dose 1 and dose 6

Dose	Measure	Dose 1				Dose 6			
		*T*_max_ (h)	*C*_max_ (pg/mL)	AUC_0–4_ (pg/mL*h)	AUC_inf_ (pg/mL)*	*T*_max_ (h)	*C*_max_ (pg/mL)	AUC_0–4_ (pg/mL*h)	AUC_inf_ (pg/mL)*
LSD 10 μg (*N* = 7)	Mean	0.5	305	891	3195	0.5	309	876	3163
	Median	0.5	324	891	3391	0.5	323	876	2928
	SD	0	68	104	454	0	48	97	609
	CV (%)	0	22	12	14	0	16	11	19
	Minimum	0.5	207	818	2516	0.5	217	807	2638
	Maximum	0.5	398	965	3729	0.5	349	945	4218
	Nbr Obs	7	7	2	7	7	7	2	7
LSD 20 μg (*N* = 8)	Mean	0.5	440	1407	4466	0.69	504	1523	5090
	Median	0.5	456	1437	4557	0.5	489	1377	5194
	SD	0	79	183	948	0.5	200	509	1537
	CV (%)	0	18	13	21	77	40	33	30
	Minimum	0.5	270	1130	2976	0.5	219	777	3241
	Maximum	0.5	537	1598	5658	2	836	2288	7837
	Nbr Obs	8	8	5	8	8	8	7	8

*% extrapolated was > 20%

### Pharmacodynamics

The one-way ANOVA analysis followed by Bonferroni pots hoc test on the CANTAB assessments (i.e., RTI, PAL, SWM, RVP) did not reveal any significant differences between treatment groups at baseline (i.e., prior to dosing), at dose 3, at dose 6, or during follow-up. Means and standard deviations, *F* statistic, and *p* value, for each variable, by dose group and day of assessments are presented in the supplementary material. Data sets with *F* statistic below the unity and *p* value above 0.05 were considered non-significant.

Repeated dosing 5 μg, 10 μg, and 20 μg of LSD did not impair balance or proprioception. An RMMM analysis conducted for these two assessments separately found no statistically significant treatment effect or interactions.

The *E*_max_ values of the subjective effects VAS and the 5D-ASC assessments were stable across repeated measures as determined by 2-way mixed-model ANOVA (*p* > 0.05). A single dimension from the 5D-ASC (i.e., “vigilance reduction”) and three questions from the subjective drug effects VAS exhibited a statistically significant linear relationship with LSD dose, shown in Table 4. The *R*^2^ coefficient for the *x*-variable in the linear relationship below indicates an increase in score with increasing dose.

**Table 4:** Drug effect variables exhibiting a statistically (*p* < 0.05) significant linear relationship with dose

	VIR (± SD)	Do you feel bad drug effects? (± SD)	Do you feel dizzy? (± SD)	Does your body feel different or changed? (± SD)
Placebo	4.69 ± 11.41	9.04 ± 18.67	2.47 ± 8.89	5.03 ± 13.59
LSD 5 μg	7.82 ± 17.90	12.76 ± 21.78	6.31 ± 16.51	10.11 ± 21.81
LSD 10 μg	9.77 ± 12.94	17.63 ± 24.42	5.62 ± 12.78	8.66 ± 18.35
LSD 20 μg	15.97 ± 17.67	27.25 ± 31.72	13.13 ± 22.32	20.65 ± 24.69
Linear relationship	*y* = 0.51*x* + 4.95	*y* = 0.894*x* + 8.98	*y* = 0.519*x* + 2.48	*y* = 0.738*x* + 4.60

The averages and standard deviations were calculated based on the scores for these measures on each of the six dosing days. The linear relationship was calculated based on these averages

## Discussion

This study examined the safety, tolerability, and PK of low doses of LSD in older healthy volunteers. Overall, results suggest that administration of low dose LSD carried no safety risk and was well tolerated during the limited 21-day period studied. Evaluation of cognitive and behavioral outcomes indicates a favorable safety profile overall, further supporting the feasibility of periodic LSD administration up to 20 μg.

The only minor clinical difference between placebo and active treatment groups was the number of headaches reported. Headaches were mild or moderate in intensity, suggesting they would not impede daily activities. Previous studies on classic serotonergic hallucinogens have also reported transient headaches (see Johnson et al. 2012).

The placebo group had a remarkably high number of nervous system and psychiatric TEAEs. One interpretation of these results is that LSD’s well-known profile created an expectancy bias that may have been a relevant factor (Polito and Stevenson 2019).

Data from other safety assessments performed, including blood pressure, pulse rate, clinical laboratory evaluations (i.e., hematology, blood chemistry, urinalysis), electrocardiogram (ECG) parameters, and physical examinations, further support the conclusion that periodic low dose LSD did not present a safety concern during the 21-day period studied. In future studies, evaluation of acute drug effect on the QT interval will be planned to address concerns over potential 5-HT_2A_ receptor agonist-mediated ECG abnormalities (Nagatomo et al. 2004). Drugs like norfenfluramine that have high affinity (Ki) and agonist potency (e.g., in phosphatidylinositol hydrolysis assays) at 5-HT_2B_ over 5-HT_2A_ receptors are known inducers of heart valve disease (HVD) (for review, see Hutcheson et al. 2011). However, drug-induced 5-HT_2B_-mediated HVD does not occur after acute dosing and only becomes observable after several months of chronic administration (Elangbam 2010). LSD is an agonist at both 5-HT_2A_R and 5-HT_2B_R, with a preferential affinity for 5-HT_2A_R over 5-HT_2B_R (based on Ki determination; IUPHAR/PBS). Therefore, in trials of longer duration involving chronic dosing, it may be important to monitor drug plasma levels to ensure adequate safety margins relative to 5-HT_2B_R engagement.

PK evaluation of low dose LSD showed that drug levels were detected following single (dose 1) and after repeated (dose 6) administration for both 10 μg and 20 μg LSD but not for 5 μg LSD. Additionally, drug levels were below LLOQ for 1 of the 8 volunteers in the 10 μg LSD group. Drug levels returned to below LLOQ prior to the last sampling time for each subject and each visit. Therefore, following repeat dosing, no sustained circulating levels of LSD above 200 pg/mL occurred with doses up to 20 μg of LSD over the 3-week period of dosing.

The *C*_max_, AUC_0–4_, and *T*_max_ had moderate variability for the 10 μg LSD group for dose 1 and dose 6, as well as the 20 μg LSD group at dose 1, which indicates that absorption was overall consistent across volunteers. The 20 μg LSD group at dose 6 displayed higher variability among subjects. Although interpretation is limited given the data set, this observation suggests that repeated administration of LSD, at the contrary of one single dose, could yield less consistent drug levels in between individual subjects over time, a possible effect that will be further explored in future trials.

Cognition tests administered via the CANTAB battery revealed no significant treatment effects for any dose group on the two dose days it was administered (i.e., doses 3 and 6). These results are consistent with recent findings that cognition was unaffected after acute dosing of 6.5 μg, 13 μg, and 26 μg LSD in healthy participants (Bershad et al. 2019). In a previous study presenting other PD results from the current trial, Yanakieva et al. (2018) argue that temporal dilation of suprasecond intervals observed under microdoses of LSD may be the result of a disruption of cognitive functions such as working memory and attention. CANTAB tests used in this study, PAL, SWM, and RVP, measure visual memory, spatial working memory, and visual attention, respectively. As none of these measures were different from placebo on dose days, the hypothesis that temporal dilation is due to a disruption of cognitive faculties does not hold. The alterations in temporal reproduction must be due to other factors that are beyond the scope of this paper.

The overall lack of detectable cognitive effects (either positive or negative) produced by LSD suggests that the doses used up to 20 μg were either insufficient to produce any discernible acute or cumulative effects in healthy participants or that doses of LSD ranging from 5 to 20 μg do not have an effect on cognition in a healthy population. Bershad et al. (2019) also found no cognitive effects of acute 6.5 μg, 13 μg, or 26 μg LSD in healthy participants. Potential effects on memory and learning should be explored in a clinical population that would not be prone to ceiling effects. While these results contradict reports of enhanced cognition in the context of recreational use (Fadiman 2011; Kuypers et al. 2019), it should be noted that not all pharmaceutical drugs that target cognitive and behavioral impairments have nootropic effects on healthy participants. For example, atomoxetine for ADHD (Bidwell et al. 2011) and memantine for AD (Repantis et al. 2010) do not enhance cognition in non-impaired individuals.

While there was no dose-response relationship with cognitive, balance, or proprioceptive tasks, regression analysis on the average ratings of the 5D-ASC showed a positive linear relationship between dose and vigilance reduction. The vigilance reduction dimension of the 5D-ASC includes statements such as “My thoughts and actions were slowed down,” “I felt sleepy,” and “I was on the verge of fainting.” One possible interpretation of these results is that participants felt more fatigued by the setting of the study under the influence of LSD, as they were sitting in their beds the whole day. Supporting this interpretation, a review of historical LSD research (1950–1970) indicates that study results appear to be highly influenced by the setting of the study (Hartogsohn 2016), and recent research has shown heightened suggestibility under the drug (Carhart-Harris et al. 2015) that may enhance the suggestion of “feeling sleepy” as the participants were confined to a bed throughout the day. Furthermore, the increase in vigilance reduction was not observed in participants in Bershad et al. (2019), discussed below. However, vigilance reduction has been reported in higher dose studies of LSD (Liechti et al. 2017; Schmid et al. 2015), and may be a dose-independent subjective effect of the drug.

Interestingly, the reduction in vigilance did not affect performance on the cognitive tasks discussed above. This lack of impairment suggests that the reduction in vigilance was tolerated by the participants, although further tests will need to be carried out to determine whether the reduction in vigilance entails a lifestyle burden for potential patients.

No linear relationship between dose and “drug liking” was found across dose days as measured in the subjective drug effects, suggesting no drug abuse potential but also no disliking. However, a positive linear relationship between the dose and “bad drug effects” was detected, as well as for “feeling dizzy” and “feeling like the body is different or changed.” In a study on younger healthy participants, Bershad et al. (2019) found a dose-dependent increase in “feel drug,” “like drug,” “feel high,” and “dislike drug,” and also reported increases in the following from the eleven new subscales on LSD dosing days compared with placebo: experience of unity, blissful state, and impaired control and cognition. An important difference between the current population and that of Bershad et al. (2019) is that the participants in our trial were psychedelic-naïve while those in the recent study had moderate previous drug use, which included at least one use of a classic psychedelic or MDMA. It could be that participants in Bershad et al. (2019) showed a propensity towards an enjoyment of the subjective effects of this class of substance. As they were likely familiar with the subjective effects of LSD, they may have been more acutely aware of smaller changes relative to a psychedelic-naïve population.

Future studies may focus on the context-dependency and qualitative nature of the bad drug effects found in the present study, and particularly better understanding whether these effects interfere with daily activities, including operating heavy machinery (i.e., driving). Questions and tasks should focus on answering what specifically these bad drug effects are, and how they may be mitigated in the context of clinical development. Furthermore, future trials may investigate intermittent administration of low doses of LSD up to 20 μg over a longer period of time.

## Conclusion

This is the first phase 1, double-blind, placebo-controlled, randomized study of the effects of LSD of 5 μg, 10 μg, or 20 μg repeatedly administered every 4 days for 21 days in healthy humans. Results yielded reassuring data regarding the safety of low dose LSD in an older healthy population. While no impairment of cognitive function was observed, a dose-dependent increase in vigilance reduction, “feeling bad drug effects,” “feeling dizzy,” and “sleepy” suggests that vigilance and alertness will need to be further explored in future trials.

These findings support the feasibility of using intermittent low dose LSD treatment in clinical therapeutic strategies and open the door for larger studies designed to evaluate anxiolytic, antidepressant, pro-cognitive, and anti-inflammatory efficacy in a clinical population, including specific evaluation as a disease-modifying therapeutic approach to treat Alzheimer’s disease.

## Electronic supplementary material

ESM 1 (PDF 175 kb)
