# Supplementary material for: Safety, tolerability, pharmacokinetics, and pharmacodynamics of low dose lysergic acid diethylamide (LSD) in healthy older volunteers
Source: Psychopharmacology (Berl). 2019 Dec 18;237(3):841–53. doi: 10.1007/s00213-019-05417-7 (PMC7036065; doi:10.1007/s00213-019-05417-7)
Supplement: Supplementary file 1 — (PDF 175 kb) [file 213_2019_5417_MOESM1_ESM.pdf]

Supplementary Material

CANTAB assessments: Means ± SD, F-statistic, and p-value, for each variable, by dose group and day of assessments

	Baseline				Dose 1			
	Placebo	5 u	10 u	20 u	Placebo	5 u	10 u	20 u
PAL Total errors (adjusted)	22.25 ± 12.77	23.17 ± 14.61	25.42 ± 16.47	23.08 ± 16.50	17.50 ± 14.95	12.17 ± 6.15	22.27 ± 14.85	17.58 ± 11.56
PAL Total attempts 8 patterns	2.58 ± 1.73	2.17 ± 1.75	2.17 ± 1.75	2.17 ± 1.80	2.08 ± 1.44	3.17 ± 0.94	3.00 ± 1.55	3.25 ± 1.22
PAL Total attempts 6 patterns	2.83 ± 0.94	2.92 ± 1.08	3.00 ± 1.21	3.00 ± 1.13	2.58 ± 1.08	2.17 ± 0.94	2.27 ± 1.27	2.25 ± 0.87
PAL Total attempts 4 patterns	1.25 ± 0.45	1.50 ± 0.52	1.50 ± 0.67	1.67 ± 0.89	1.17 ± 0.39	1.67 ± 0.78	1.82 ± 0.98	1.83 ± 0.94
PAL Total attempts 2 patterns	1.17 ± 0.39	1.08 ± 0.29	1.17 ± 0.39	1.08 ± 0.29	1.00 ± 0.00	1.00 ± 0.00	1.09 ± 0.30	1.08 ± 0.29
PAL Total errors 8 patterns	9.25 ± 8.30	6.42 ± 7.82	7.00 ± 7.83	5.42 ± 6.82	5.58 ± 6.92	8.42 ± 5.84	9.91 ± 7.26	10.25 ± 6.22
PAL Total errors 6 patterns	5.17 ± 3.88	6.25 ± 4.67	7.67 ± 6.02	7.08 ± 5.11	4.50 ± 4.81	2.50 ± 2.20	3.55 ± 3.39	3.50 ± 3.87
PAL Total errors 4 patterns	0.58 ± 1.08	1.08 ± 1.38	1.17 ± 2.08	1.17 ± 1.75	0.42 ± 1.00	1.25 ± 1.42	1.73 ± 2.49	1.33 ± 1.56
PAL Total errors 2 patterns	0.25 ± 0.62	0.08 ± 0.29	0.25 ± 0.62	0.08 ± 0.29	0.00 ± 0.00	0.00 ± 0.00	0.18 ± 0.60	0.17 ± 0.58
PAL Total errors	15.25 ± 7.91	13.83 ± 8.42	16.08 ± 7.23	13.75 ± 7.53	10.50 ± 6.35	12.17 ± 6.15	15.36 ± 6.93	15.25 ± 7.71
PAL Total errors 8 shapes adjusted	16.25 ± 9.38	15.75 ± 10.98	16.33 ± 10.43	14.75 ± 11.23	12.58 ± 11.09	8.42 ± 5.84	15.00 ± 8.37	12.58 ± 7.20
PAL Total errors 6 shapes adjusted	5.17 ± 3.88	6.25 ± 4.67	7.67 ± 6.02	7.08 ± 5.11	4.50 ± 4.81	2.50 ± 2.20	5.36 ± 5.80	3.50 ± 3.87
PAL Total errors 4 shapes adjusted	0.58 ± 1.08	1.08 ± 1.38	1.17 ± 2.08	1.17 ± 1.75	0.42 ± 1.00	1.25 ± 1.42	1.73 ± 2.49	1.33 ± 1.56
PAL Total errors 2 shapes adjusted	0.25 ± 0.62	0.08 ± 0.29	0.25 ± 0.62	0.08 ± 0.29	0.00 ± 0.00	0.00 ± 0.00	0.18 ± 0.60	0.17 ± 0.58
PAL Total Number of Patterns reached	7.50 ± 0.90	7.33 ± 0.98	7.33 ± 0.98	7.33 ± 0.98	7.50 ± 0.90	8.00 ± 0.00	7.45 ± 1.29	7.83 ± 0.58
PAL Mean attempts to success	2.33 ± 1.87	1.58 ± 1.38	1.92 ± 1.51	1.83 ± 1.27	1.08 ± 0.90	2.50 ± 1.17	1.73 ± 1.19	2.42 ± 0.79
PAL First attempt memory score	10.58 ± 3.37	11.33 ± 3.87	10.00 ± 4.49	10.67 ± 4.12	13.08 ± 3.50	13.08 ± 2.61	10.27 ± 4.43	11.58 ± 3.68
PAL Total attemps	7.83 ± 1.64	7.67 ± 1.78	7.83 ± 1.53	7.92 ± 1.51	6.83 ± 1.19	8.00 ± 1.60	8.18 ± 1.72	8.42 ± 1.73
RTI Mean five-choice reaction time (ms)	546.03 ± 56.84	563.45 ± 50.93	601.81 ± 119.90	542.32 ± 74.48	534.57 ± 33.60	560.85 ± 81.82	517.53 ± 84.29	545.81 ± 66.59
RTI Five choice error score (all)	2.83 ± 2.17	3.75 ± 2.99	4.25 ± 5.31	3.42 ± 2.71	2.92 ± 3.60	5.08 ± 3.78	3.00 ± 2.86	3.00 ± 2.30
RTI Five choice error score (no response)	0.08 ± 0.29	0.17 ± 0.39	0.17 ± 0.58	0.08 ± 0.29	0.17 ± 0.39	0.17 ± 0.39	0.09 ± 0.30	0.17 ± 0.39
RTI Five choice error score (premature)	1.75 ± 1.96	2.17 ± 2.86	2.42 ± 4.06	1.83 ± 1.90	1.58 ± 2.39	2.83 ± 4.13	1.73 ± 2.61	1.33 ± 1.23
RTI Five choice error score (inaccurate)	0.50 ± 0.52	0.75 ± 0.62	0.33 ± 0.65	1.17 ± 1.53	0.50 ± 0.90	1.08 ± 1.56	0.64 ± 0.92	1.33 ± 1.78
RTI Five choice error score (incorrect)	0.08 ± 0.29	0.00 ± 0.00	0.00 ± 0.00	0.00 ± 0.00	0.00 ± 0.00	0.00 ± 0.00	0.09 ± 0.30	0.00 ± 0.00
RTI Five choice movement time (SD)	63.42 ± 51.93	55.32 ± 20.36	83.43 ± 70.15	57.98 ± 37.92	75.52 ± 63.28	62.00 ± 28.24	76.05 ± 48.96	60.40 ± 38.07
RTI Five choice movement time (ms)	304.42 ± 91.58	261.54 ± 63.11	314.67 ± 103.44	266.88 ± 74.23	330.38 ± 116.04	293.96 ± 52.46	340.27 ± 141.18	273.71 ± 89.58
RTI Five choice movement time (ms)	310.71 ± 85.91	268.33 ± 57.53	327.17 ± 111.45	269.67 ± 71.46	342.67 ± 128.18	297.38 ± 56.41	349.75 ± 147.94	275.95 ± 86.07
RTI Five choice reaction time (ms)	60.73 ± 26.68	63.71 ± 27.35	132.25 ± 137.81	77.37 ± 33.08	70.46 ± 26.74	66.43 ± 19.97	75.18 ± 46.15	73.89 ± 41.82

Supplementary Material

CANTAB assessments: Means ± SD, F-statistic, and p-value, for each variable, by dose group and day of assessments

	Dose 6				Follow-up				One-way ANOVA	Bonferroni post-hoc
	Placebo	5 u	10 u	20 u	Placebo	5 u	10 u	20 u		
PAL Total errors (adjusted)	18.75 ± 15.17	16.67 ± 13.41	24.64 ± 19.12	24.25 ± 15.53	20.67 ± 17.65	15.67 ± 11.19	27.58 ± 16.10	13.58 ± 11.70	F= 1.24, p= 0.23	ns
PAL Total attempts 8 patterns	1.75 ± 1.60	2.50 ± 1.57	2.18 ± 1.83	1.67 ± 1.83	2.17 ± 1.59	3.08 ± 1.38	2.08 ± 1.93	2.75 ± 1.29	F= 1.03, p= 0.43	ns
PAL Total attempts 6 patterns	3.08 ± 0.90	2.42 ± 1.24	2.36 ± 1.36	2.75 ± 1.42	2.67 ± 0.98	2.50 ± 1.00	3.00 ± 1.28	2.42 ± 1.00	F= 1.08, p= 0.38	ns
PAL Total attempts 4 patterns	1.33 ± 0.49	1.33 ± 0.65	1.64 ± 0.92	1.42 ± 0.79	2.08 ± 1.16	1.42 ± 0.51	1.58 ± 0.67	1.67 ± 0.78	F= 1.12, p= 0.34	ns
PAL Total attempts 2 patterns	1.00 ± 0.00	1.08 ± 0.29	1.09 ± 0.30	1.17 ± 0.39	1.08 ± 0.29	1.00 ± 0.00	1.00 ± 0.00	1.17 ± 0.39	F= 0.71, p= 0.80	ns
PAL Total errors 8 patterns	3.17 ± 5.08	6.67 ± 6.68	5.45 ± 5.34	3.50 ± 3.90	6.33 ± 6.27	9.25 ± 7.88	6.42 ± 6.46	5.83 ± 4.30	F= 1.14, p= 0.31	ns
PAL Total errors 6 patterns	5.67 ± 3.92	4.67 ± 4.54	5.09 ± 5.19	5.58 ± 4.87	5.25 ± 5.22	3.33 ± 3.42	8.25 ± 7.07	3.92 ± 4.27	F= 1.49, p= 0.09	ns
PAL Total errors 4 patterns	0.58 ± 1.16	0.58 ± 1.24	2.00 ± 4.40	0.92 ± 1.68	1.92 ± 2.50	0.75 ± 1.06	1.25 ± 1.36	1.25 ± 1.60	F= 0.75, p= 0.76	ns
PAL Total errors 2 patterns	0.00 ± 0.00	0.08 ± 0.29	0.09 ± 0.30	0.25 ± 0.62	0.17 ± 0.58	0.00 ± 0.00	0.00 ± 0.00	0.25 ± 0.62	F= 0.68, p= 0.84	ns
PAL Total errors	9.42 ± 5.78	12.00 ± 7.80	12.64 ± 4.90	10.25 ± 3.84	13.67 ± 8.04	13.33 ± 7.51	15.92 ± 7.01	11.25 ± 7.25	F= 1.51, p= 0.08	ns
PAL Total errors 8 shapes adjusted	12.50 ± 12.30	11.33 ± 9.77	15.64 ± 10.29	17.50 ± 11.05	13.33 ± 10.15	11.58 ± 8.96	18.08 ± 9.29	8.17 ± 7.36	F= 1.11, p= 0.35	ns
PAL Total errors 6 shapes adjusted	5.67 ± 3.92	4.67 ± 4.54	6.91 ± 6.55	5.58 ± 4.87	5.25 ± 5.22	3.33 ± 3.42	8.25 ± 7.07	3.92 ± 4.27	F= 1.36, p= 0.15	ns
PAL Total errors 4 shapes adjusted	0.58 ± 1.16	0.58 ± 1.24	2.00 ± 4.40	0.92 ± 1.68	1.92 ± 2.50	0.75 ± 1.06	1.25 ± 1.36	1.25 ± 1.60	F= 0.75, p= 0.76	ns
PAL Total errors 2 shapes adjusted	0.00 ± 0.00	0.08 ± 0.29	0.09 ± 0.30	0.25 ± 0.62	0.17 ± 0.58	0.00 ± 0.00	0.00 ± 0.00	0.25 ± 0.62	F= 0.68, p= 0.84	ns
PAL Total Number of Patterns reached	7.33 ± 0.98	7.67 ± 0.78	7.09 ± 1.38	7.00 ± 1.04	7.50 ± 0.90	7.83 ± 0.58	7.17 ± 1.03	7.83 ± 0.58	F= 1.04, p= 0.42	ns
PAL Mean attempts to success	1.00 ± 1.28	2.00 ± 1.21	1.45 ± 1.44	1.33 ± 1.37	2.17 ± 1.34	1.75 ± 1.06	1.83 ± 1.34	2.50 ± 1.57	F= 1.40, p= 0.13	ns
PAL First attempt memory score	13.00 ± 4.29	12.58 ± 3.42	10.55 ± 4.48	11.00 ± 3.86	10.92 ± 4.68	12.42 ± 3.00	9.75 ± 3.39	12.50 ± 4.19	F= 1.22, p= 0.25	ns
PAL Total attemps	7.17 ± 1.75	7.33 ± 1.44	7.27 ± 1.85	7.00 ± 1.71	8.00 ± 1.86	8.00 ± 1.41	7.67 ± 2.02	8.00 ± 1.71	F= 0.82, p= 0.69	ns
RTI Mean five-choice reaction time (ms)	567.41 ± 58.31	526.08 ± 68.76	549.48 ± 58.77	533.40 ± 46.65	576.39 ± 62.97	543.04 ± 47.38	582.15 ± 79.63	547.14 ± 79.94	F= 1.24, p= 0.23	ns
RTI Five choice error score (all)	1.42 ± 1.68	3.25 ± 6.05	1.82 ± 1.72	2.83 ± 2.08	2.42 ± 2.15	2.67 ± 2.67	6.00 ± 8.15	2.42 ± 2.43	F= 1.08, p= 0.38	ns
RTI Five choice error score (no response)	0.00 ± 0.00	0.00 ± 0.00	0.00 ± 0.00	0.08 ± 0.29	0.00 ± 0.00	0.00 ± 0.00	0.00 ± 0.00	0.00 ± 0.00	F= 1.01, p= 0.45	ns
RTI Five choice error score (premature)	0.83 ± 1.27	1.92 ± 5.71	1.00 ± 1.55	1.17 ± 1.53	1.83 ± 1.85	1.58 ± 2.43	3.67 ± 6.07	1.33 ± 2.23	F= 0.66, p= 0.86	ns
RTI Five choice error score (inaccurate)	0.17 ± 0.39	0.75 ± 0.97	0.18 ± 0.40	1.00 ± 1.04	0.17 ± 0.58	0.58 ± 0.67	0.75 ± 1.42	0.92 ± 1.56	F= 1.12, p= 0.34	ns
RTI Five choice error score (incorrect)	0.00 ± 0.00	0.00 ± 0.00	0.00 ± 0.00	0.00 ± 0.00	0.08 ± 0.29	0.00 ± 0.00	0.00 ± 0.00	0.00 ± 0.00	F= 0.92, p= 0.56	ns
RTI Five choice movement time (SD)	111.15 ± 169.41	47.46 ± 17.15	74.11 ± 44.67	51.56 ± 24.10	61.79 ± 24.60	64.86 ± 27.28	79.57 ± 63.44	56.80 ± 31.99	F= 0.80, p= 0.71	ns
RTI Five choice movement time (ms)	308.50 ± 87.18	291.83 ± 66.13	328.55 ± 95.04	285.83 ± 60.61	314.42 ± 110.24	290.54 ± 65.66	360.54 ± 129.08	274.83 ± 67.57	F= 1.22, p= 0.25	ns
RTI Five choice movement time (ms)	327.38 ± 96.77	295.30 ± 67.10	337.27 ± 99.35	288.61 ± 58.51	321.49 ± 102.28	293.63 ± 61.55	374.67 ± 151.94	275.97 ± 66.08	F= 1.40, p= 0.13	ns
RTI Five choice reaction time (ms)	63.93 ± 23.47	53.89 ± 14.18	66.27 ± 27.59	58.29 ± 21.11	62.72 ± 23.01	67.08 ± 15.49	93.46 ± 92.87	55.72 ± 17.42	F= 1.35, p= 0.15	ns

Supplementary Material

CANTAB assessments: Means ± SD, F-statistic, and p-value, for each variable, by dose group and day of assessments

	Baseline				Dose 1			
	Placebo	5 u	10 u	20 u	Placebo	5 u	10 u	20 u
RTI Five choice reaction time (ms)	534.75 ± 60.44	553.71 ± 53.65	564.17 ± 82.76	530.79 ± 69.27	527.33 ± 35.93	551.58 ± 80.07	504.95 ± 76.42	529.58 ± 58.16
RVP Mean response latency (ms)	481.75 ± 69.99	533.71 ± 68.43	579.29 ± 177.80	496.94 ± 63.55	479.90 ± 46.42	522.07 ± 78.64	511.87 ± 112.21	520.62 ± 94.67
RVP Total misses	17.42 ± 6.54	18.00 ± 8.24	17.25 ± 10.18	18.17 ± 9.05	14.08 ± 7.37	13.08 ± 8.87	17.45 ± 12.41	17.83 ± 9.62
RVP Probability of false alarm	0.01 ± 0.01	0.01 ± 0.01	0.02 ± 0.02	0.05 ± 0.13	0.01 ± 0.01	0.01 ± 0.01	0.01 ± 0.01	0.02 ± 0.03
RVP Probability of hit	0.68 ± 0.12	0.67 ± 0.15	0.68 ± 0.19	0.66 ± 0.17	0.74 ± 0.14	0.76 ± 0.16	0.68 ± 0.23	0.67 ± 0.18
RVP Total False alarms	7.08 ± 5.99	3.92 ± 3.87	7.75 ± 9.73	26.08 ± 66.93	6.42 ± 7.33	3.83 ± 3.74	6.91 ± 6.83	9.75 ± 13.75
RVP Total hits	36.58 ± 6.54	36.00 ± 8.24	36.75 ± 10.18	35.83 ± 9.05	39.92 ± 7.37	40.92 ± 8.87	36.55 ± 12.41	36.17 ± 9.62
RVP A	0.91 ± 0.04	0.91 ± 0.04	0.91 ± 0.06	0.90 ± 0.04	0.93 ± 0.04	0.94 ± 0.04	0.91 ± 0.07	0.91 ± 0.05
RVP Response latency (SD) (ms)	137.43 ± 95.98	193.71 ± 61.76	194.53 ± 110.38	151.59 ± 84.89	139.07 ± 90.58	191.41 ± 83.81	176.34 ± 128.24	186.90 ± 124.38
RVP Median response latency (ms)	449.96 ± 59.73	476.67 ± 45.35	534.21 ± 173.14	465.13 ± 43.53	450.54 ± 62.21	465.21 ± 57.58	456.41 ± 74.00	468.17 ± 61.70
SWM Total errors	10.08 ± 7.93	11.83 ± 9.09	17.00 ± 10.15	16.08 ± 8.97	11.25 ± 7.75	9.50 ± 7.27	18.82 ± 7.80	11.58 ± 8.87
SWM Problem Reached	5.00 ± 0.00	5.00 ± 0.00	5.00 ± 0.00	5.00 ± 0.00	5.00 ± 0.00	5.00 ± 0.00	5.00 ± 0.00	5.00 ± 0.00
SWM Strategy (6-8 boxes)	8.33 ± 2.53	8.92 ± 1.93	8.50 ± 2.28	8.33 ± 2.06	7.83 ± 3.16	8.17 ± 1.70	9.00 ± 2.68	8.50 ± 2.50
SWM Double errors 8 boxes	0.17 ± 0.39	0.33 ± 0.65	0.92 ± 2.27	0.58 ± 0.90	0.08 ± 0.29	0.33 ± 0.89	1.64 ± 2.38	0.67 ± 1.78
SWM Within errors 8 boxes	0.25 ± 0.62	0.42 ± 0.79	1.50 ± 2.84	1.17 ± 1.47	0.50 ± 0.80	0.42 ± 1.00	2.64 ± 3.07	0.83 ± 2.04
SWM Between errors 8 boxes	6.08 ± 5.40	7.42 ± 5.79	10.67 ± 6.04	11.75 ± 6.05	6.67 ± 5.47	6.42 ± 5.43	12.73 ± 5.44	7.50 ± 6.78
SWM Total errors 8 boxes	6.17 ± 5.51	7.50 ± 5.85	11.25 ± 6.55	12.33 ± 5.97	7.08 ± 5.38	6.50 ± 5.49	13.73 ± 5.41	7.67 ± 6.93
SWM Double errors 6 boxes	0.08 ± 0.29	0.08 ± 0.29	0.08 ± 0.29	0.17 ± 0.39	0.17 ± 0.39	0.00 ± 0.00	0.09 ± 0.30	0.25 ± 0.87
SWM Within errors 6 boxes	0.17 ± 0.39	0.17 ± 0.58	0.17 ± 0.58	0.25 ± 0.62	0.25 ± 0.62	0.08 ± 0.29	0.27 ± 0.47	0.25 ± 0.87
SWM Between errors 6 boxes	3.25 ± 3.17	3.92 ± 3.29	4.83 ± 3.04	3.08 ± 3.06	3.50 ± 3.21	2.33 ± 3.17	4.27 ± 3.98	3.50 ± 3.90
SWM Total errors 6 boxes	3.33 ± 3.11	4.00 ± 3.44	4.92 ± 3.20	3.17 ± 3.24	3.58 ± 3.26	2.42 ± 3.32	4.45 ± 3.93	3.50 ± 3.90
SWM Double errors 4 boxes	0.00 ± 0.00	0.00 ± 0.00	0.00 ± 0.00	0.00 ± 0.00	0.00 ± 0.00	0.00 ± 0.00	0.00 ± 0.00	0.00 ± 0.00
SWM Within errors 4 boxes	0.00 ± 0.00	0.00 ± 0.00	0.00 ± 0.00	0.00 ± 0.00	0.00 ± 0.00	0.00 ± 0.00	0.00 ± 0.00	0.00 ± 0.00
SWM Between errors 4 boxes	0.58 ± 1.38	0.33 ± 0.78	0.83 ± 0.94	0.58 ± 1.00	0.58 ± 1.24	0.58 ± 1.38	0.64 ± 1.12	0.42 ± 0.67
SWM Total errors 4 boxes	0.58 ± 1.38	0.33 ± 0.78	0.83 ± 0.94	0.58 ± 1.00	0.58 ± 1.24	0.58 ± 1.38	0.64 ± 1.12	0.42 ± 0.67
SWM Double errors	0.25 ± 0.45	0.42 ± 0.67	1.00 ± 2.30	0.75 ± 1.22	0.25 ± 0.45	0.33 ± 0.89	1.73 ± 2.37	0.92 ± 2.15
SWM Within errors	0.42 ± 0.67	0.58 ± 0.90	1.67 ± 2.99	1.42 ± 2.02	0.75 ± 1.06	0.50 ± 1.00	2.91 ± 3.36	1.08 ± 2.35
SWM Between errors	9.92 ± 7.97	11.67 ± 8.92	16.33 ± 9.54	15.42 ± 8.73	10.75 ± 7.71	9.33 ± 7.24	17.64 ± 7.74	11.42 ± 8.76

Supplementary Material

CANTAB assessments: Means ± SD, F-statistic, and p-value, for each variable, by dose group and day of assessments

	Dose 6				Follow-up				One-way ANOVA	Bonferroni post-hoc
	Placebo	5 u	10 u	20 u	Placebo	5 u	10 u	20 u		
RTI Five choice reaction time (ms)	550.38 ± 53.15	516.96 ± 66.58	542.36 ± 62.11	527.67 ± 44.89	571.71 ± 67.16	529.88 ± 54.06	572.38 ± 63.52	535.00 ± 79.82	F= 0.94, p= 0.53	ns
RVP Mean response latency (ms)	500.65 ± 46.88	516.84 ± 77.94	480.82 ± 97.27	502.25 ± 67.73	523.58 ± 107.98	513.62 ± 54.61	499.08 ± 107.63	491.43 ± 54.45	F= 1.44, p= 0.11	ns
RVP Total misses	13.17 ± 8.09	12.25 ± 6.89	14.36 ± 14.33	16.42 ± 11.04	16.75 ± 13.74	8.75 ± 6.14	15.00 ± 13.93	14.25 ± 8.81	F= 1.54, p= 0.07	ns
RVP Probability of false alarm	0.02 ± 0.02	0.01 ± 0.01	0.01 ± 0.01	0.01 ± 0.01	0.01 ± 0.01	0.01 ± 0.01	0.01 ± 0.01	0.01 ± 0.01	F= 1.05, p= 0.41	ns
RVP Probability of hit	0.76 ± 0.15	0.77 ± 0.13	0.73 ± 0.27	0.70 ± 0.20	0.69 ± 0.25	0.84 ± 0.11	0.72 ± 0.26	0.74 ± 0.16	F= 1.54, p= 0.07	ns
RVP Total False alarms	8.00 ± 9.36	3.25 ± 4.37	5.09 ± 3.65	5.58 ± 4.68	4.92 ± 5.04	2.83 ± 3.79	5.42 ± 6.53	3.50 ± 4.03	F= 1.03, p= 0.42	ns
RVP Total hits	40.83 ± 8.09	41.75 ± 6.89	39.64 ± 14.33	37.58 ± 11.04	37.25 ± 13.74	45.25 ± 6.14	39.00 ± 13.93	39.75 ± 8.81	F= 1.54, p= 0.07	ns
RVP A	0.93 ± 0.04	0.94 ± 0.03	0.93 ± 0.07	0.92 ± 0.06	0.92 ± 0.06	0.96 ± 0.03	0.92 ± 0.07	0.93 ± 0.04	F= 1.73, p= 0.03*	ns
RVP Response latency (SD) (ms)	173.74 ± 105.98	173.22 ± 77.84	129.93 ± 68.13	153.42 ± 73.84	138.42 ± 63.52	138.97 ± 45.06	146.39 ± 83.24	140.85 ± 83.20	F= 1.28, p= 0.20	ns
RVP Median response latency (ms)	457.63 ± 55.17	472.21 ± 66.58	454.91 ± 79.92	472.50 ± 66.13	498.33 ± 114.26	479.00 ± 55.37	461.25 ± 95.71	463.33 ± 44.34	F= 1.37, p= 0.15	ns
SWM Total errors	9.42 ± 9.73	12.50 ± 7.93	13.64 ± 9.16	9.83 ± 8.13	9.50 ± 10.21	10.67 ± 8.78	14.50 ± 9.55	11.92 ± 10.04	F= 1.75, p= 0.03*	ns
SWM Problem Reached	5.00 ± 0.00	5.00 ± 0.00	5.00 ± 0.00	5.00 ± 0.00	5.00 ± 0.00	5.00 ± 0.00	5.00 ± 0.00	5.00 ± 0.00		ns
SWM Strategy (6-8 boxes)	6.33 ± 3.08	7.50 ± 2.24	7.09 ± 2.91	7.33 ± 2.90	6.58 ± 3.18	7.67 ± 2.74	8.50 ± 3.03	7.33 ± 2.74	F= 1.28, p= 0.20	ns
SWM Double errors 8 boxes	0.17 ± 0.39	0.17 ± 0.39	0.27 ± 0.47	0.17 ± 0.39	0.17 ± 0.39	0.42 ± 0.51	0.83 ± 1.19	1.00 ± 1.48	F= 1.23, p= 0.24	ns
SWM Within errors 8 boxes	0.42 ± 0.79	0.33 ± 0.89	0.55 ± 0.93	0.42 ± 0.67	0.42 ± 0.90	0.58 ± 0.79	1.33 ± 1.87	1.08 ± 1.56	F= 1.43, p= 0.11	ns
SWM Between errors 8 boxes	6.50 ± 6.86	8.92 ± 5.37	9.27 ± 6.47	5.83 ± 4.97	5.50 ± 6.52	7.50 ± 6.08	9.67 ± 6.84	8.83 ± 6.38	F= 1.85, p= 0.02*	ns
SWM Total errors 8 boxes	6.75 ± 6.84	9.08 ± 5.47	9.55 ± 6.30	6.08 ± 4.87	5.75 ± 6.34	7.67 ± 6.17	10.17 ± 7.16	8.92 ± 6.53	F= 2.04, p= 0.01**	ns
SWM Double errors 6 boxes	0.25 ± 0.45	0.00 ± 0.00	0.00 ± 0.00	0.25 ± 0.62	0.33 ± 0.49	0.00 ± 0.00	0.33 ± 0.89	0.17 ± 0.58	F= 0.78, p= 0.73	ns
SWM Within errors 6 boxes	0.42 ± 0.51	0.08 ± 0.29	0.00 ± 0.00	0.42 ± 1.00	0.58 ± 0.67	0.00 ± 0.00	0.50 ± 1.24	0.33 ± 0.89	F= 0.83, p= 0.67	ns
SWM Between errors 6 boxes	2.17 ± 2.92	3.00 ± 3.88	3.18 ± 3.40	3.25 ± 3.65	3.08 ± 3.48	2.50 ± 2.65	4.08 ± 3.09	2.75 ± 4.27	F= 1.09, p= 0.37	ns
SWM Total errors 6 boxes	2.33 ± 2.81	3.08 ± 3.96	3.18 ± 3.40	3.42 ± 3.82	3.33 ± 3.55	2.50 ± 2.65	4.25 ± 3.31	2.92 ± 4.48	F= 1.03, p= 0.43	ns
SWM Double errors 4 boxes	0.00 ± 0.00	0.00 ± 0.00	0.09 ± 0.30	0.00 ± 0.00	0.00 ± 0.00	0.00 ± 0.00	0.00 ± 0.00	0.00 ± 0.00	F= 0.98, p= 0.48	ns
SWM Within errors 4 boxes	0.00 ± 0.00	0.00 ± 0.00	0.09 ± 0.30	0.00 ± 0.00	0.00 ± 0.00	0.00 ± 0.00	0.00 ± 0.00	0.00 ± 0.00	F= 0.91, p= 0.57	ns
SWM Between errors 4 boxes	0.33 ± 1.15	0.33 ± 0.78	0.91 ± 1.30	0.33 ± 1.15	0.42 ± 1.00	0.50 ± 1.17	0.08 ± 0.29	0.08 ± 0.29	F= 0.97, p= 0.49	ns
SWM Total errors 4 boxes	0.33 ± 1.15	0.33 ± 0.78	0.91 ± 1.30	0.33 ± 1.15	0.42 ± 1.00	0.50 ± 1.17	0.08 ± 0.29	0.08 ± 0.29	F= 1.02, p= 0.43	ns
SWM Double errors	0.42 ± 0.67	0.17 ± 0.39	0.36 ± 0.50	0.42 ± 0.67	0.50 ± 0.67	0.42 ± 0.51	1.17 ± 1.34	1.17 ± 1.99	F= 1.06, p= 0.40	ns
SWM Within errors	0.83 ± 1.03	0.42 ± 0.90	0.64 ± 0.92	0.83 ± 1.03	1.00 ± 1.21	0.58 ± 0.79	1.83 ± 1.99	1.42 ± 2.39	F= 1.29, p= 0.19	ns
SWM Between errors	9.00 ± 9.79	12.25 ± 7.93	13.36 ± 9.32	9.42 ± 8.03	9.00 ± 10.35	10.50 ± 8.74	13.83 ± 9.07	11.67 ± 9.66	F= 1.65, p= 0.05*	ns
